# A band-gap database for semiconducting inorganic materials calculated with hybrid functional

**DOI:** 10.1038/s41597-020-00723-8

**Published:** 2020-11-11

**Authors:** Sangtae Kim, Miso Lee, Changho Hong, Youngchae Yoon, Hyungmin An, Dongheon Lee, Wonseok Jeong, Dongsun Yoo, Youngho Kang, Yong Youn, Seungwu Han

**Affiliations:** 1 Department of Materials Science and Engineering and Research Institute of Advanced Materials, Seoul National University, Seoul, 08826 Korea; 2 Department of Materials Science and Engineering, Incheon National University, Incheon, 22012 Korea

**Keywords:** Electronic structure, Computational methods, Electronic properties and materials, Magnetic properties and materials

## Abstract

Semiconducting inorganic materials with band gaps ranging between 0 and 5 eV constitute major components in electronic, optoelectronic and photovoltaic devices. Since the band gap is a primary material property that affects the device performance, large band-gap databases are useful in selecting optimal materials in each application. While there exist several band-gap databases that are theoretically compiled by density-functional-theory calculations, they suffer from computational limitations such as band-gap underestimation and metastable magnetism. In this data descriptor, we present a computational database of band gaps for 10,481 materials compiled by applying a hybrid functional and considering the stable magnetic ordering. For benchmark materials, the root-mean-square error in reference to experimental data is 0.36 eV, significantly smaller than 0.75–1.05 eV in the existing databases. Furthermore, we identify many small-gap materials that are misclassified as metals in other databases. By providing accurate band gaps, the present database will be useful in screening materials in diverse applications.

## Abstract (summary)

**Table:** 

Measurement(s)	band gap • semiconducting inorganic material
Technology Type(s)	computational modeling technique
Sample Characteristic - Environment	material entity

Machine-accessible metadata file describing the reported data: 10.6084/m9.figshare.13083980

## Background & Summary

The band gap (*E*_g_) is a fundamental quantity that directly relates to usability of materials in optical, electronic, and energy applications. For instance, in photovoltaic devices, materials with a direct *E*_g_ of ∼1.3 eV^1,2^, corresponding to the Shockley-Queisser limit, are favored as photo-absorbers that maximize the solar-cell efficiency. In power electronics, semiconductors with *E*_g_ ≥ 3 eV are employed to sustain high electric fields^3^. To increase the figure of merit in thermoelectric devices, materials with *E*_g_ of 10 *k*_B_*T*_op_ where *k*_B_ and *T*_op_ are the Boltzmann constant and operating temperature, respectively, are selected^4^. Given the central role of *E*_g_, a database of *E*_g_ over a wide range of materials can expedite the material selection in specific applications by factoring out suboptimal candidates rapidly. Currently, popular material databases such as the Materials Project^5^, the Automatic Flow of Materials Discovery Library (AFLOW)^6^, the Open Quantum Materials Database (OQMD)^7^, and the Joint Automated Repository for Various Integrated Simulations (JARVIS)^8^ provide theoretical *E*_g_ for up to one million inorganic materials. However, most of them were obtained by semilocal functionals with a generalized gradient approximation (GGA), which underestimates *E*_g_ by typically 30–40%^9^. (MatDB^10^ provides accurate quasi-particle band gaps, but the number of data is limited to hundreds.) To compensate for this, AFLOW provides adjusted *E*_g_ using a linear fit to experimental data^11^. However, such a universal correction may not address element-dependent error fluctuations. We note that JARVIS provides *E*_g_ based on meta-GGA^12^, which significantly improves the accuracy. As a related issue, many small-gap semiconductors such as Ge, InAs, PdO, Zn_3_As_2_, and Ag_2_O are misclassified as metals, which can affect selection of narrow-gap semiconductors in IR sensors^13^, for instance. (In JARVIS, some of these errors are resolved by meta-GGA.) Besides the underestimation of *E*_g_, all the databases consider only the ferromagnetic ordering for spin-polarized systems due to computational convenience, which can cause significant errors in *E*_g_ of antiferromagnetic materials. For instance, the antiferromagnetic NiO has an experimental *E*_g_ of 4.3 eV^14^, but the computational *E*_g_ ranges over 2.2–2.6 eV in the ferromagnetic ordering and GGA functional^5–7^ while the correct antiferromagnetic ordering produces 4.5 eV within the hybrid functional.

Addressing limitations in the existing material databases, we herein report a theoretical dataset of fundamental and optical *E*_g_ computed by employing a hybrid functional and identifying the stable magnetic ordering, thus providing more accurate *E*_g_ than the existing databases. For the high-throughput computational workflow, we employ ‘Automated *Ab initio* Modeling of Materials Property Package’ (AMP^2^)^15^, which is a fully automated package for density functional theory (DFT) calculations of crystalline properties. AMP^2^ addresses the band-gap underestimation in semilocal functionals with the help of a hybrid functional, thereby producing a more accurate *E*_g_, even if the material is incorrectly metallic within the semilocal functional. Furthermore, the package finds the antiferromagnetic ground state based on an effective Ising model. The present database focuses on materials with 0 eV < *E*_g_ < 5 eV, which covers most semiconducting materials. The target materials are selected from Inorganic Crystal Structure Database (ICSD)^16^ and partly filtered by information from the Materials Project database. In total, the database collects *E*_g_ for 10,481 materials that encompass most inorganic solids with *E*_g_ ranging between 0 and 5 eV. For 116 benchmark materials, the root-mean-square error (RMSE) with respect to experimental data is 0.36 eV, significantly smaller than 0.75–1.05 eV in the existing databases. The resulting data are available online at figshare^17^ or SNUMAT^18^.

## Methods

### High-throughput methodology: AMP^2^

The present database is constructed by employing AMP^2^ which is an automation script operating VASP^19–21^. Starting only with the initial crystalline structure, AMP^2^ provides band structure, *E*_g_, effective mass, density of states (DOS) and dielectric constants of the crystal by automatically pipelining computational procedures. To summarize computational settings relevant in the present work, we employ GGA developed by Perdew-Burke-Ernzerhof (PBE)^22^ for the exchange-correlation functional for structural relaxation and identifying band edges. The *E*_g_ is obtained by ‘one-shot’ hybrid functional (specifically, HSE06^23^ (simply HSE hereafter)) calculations in which the package estimates *E*_g_ from HSE eigenvalues at **k** points of band edges found with PBE (crystal structures are also fixed to those relaxed by PBE). In the previous study^24^, it was demonstrated that band edges from PBE and HSE lie at the same **k** points, which is confirmed again in the present work with Si, SrS, BAs, BeS, AlAs, AgI, AgGaTe_2_, ZnSiAs_2_, and ZnIn_2_Se_4_. In addition, the small structural differences between PBE and HSE^25^ would not affect the band gap significantly, except for small-gap semiconductors (see below). (This is also the case for systems that go from metallic in PBE to insulating in HSE.) This supports that the one-shot scheme can produce *E*_g_ close to the full hybrid calculations. If the material is identified as a metal within PBE, AMP^2^ inspects DOS, and if DOS at the Fermi level normalized by the valence band (*D*_F_/*D*_VB_) is less than a threshold, the package further tests a possible gap-opening by the one-shot hybrid calculation. The PBE+*U* method is applied on 3d orbitals^26^ only when the material has a finite *E*_g_. About pseudopotentials, we mostly employ those without any tags in the VASP database, which tends to reduce the number of valence electrons. For further details, we refer to the original publication^15^.

Computational parameters used in the present work follow the default setting of AMP^2^ except that the package applies the PBE+*U* method on Ce 4f levels with the *U* value of 4 eV^27^. (The pseudopotentials for La and Ce treat f levels as valence.) Furthermore, for compounds including Tl, Pb, and Bi, *E*_g_ is recalculated by including the spin-orbit coupling (SOC) when the default *E*_g_ without SOC is smaller than 1 eV. (The band edges are also resought with SOC.) This is because typical SOC corrections of ∼0.5 eV would be critical in these cases.

In identifying the stable collinear magnetic ordering, AMP^2^ applies a genetic algorithm to the Ising model^28^. This approach finds the stable magnetic ordering correctly for many compounds. However, the original formulation requires a large supercell to isolate exchange interactions from periodic images, which costs significant computational resources and also suffers from ill-convergence in electronic iterations. To resolve this, we here develop an alternative method in obtaining exchange parameters. First, we choose a minimal supercell under the following two conditions: (i) A magnetic site α and its periodic images in other supercells are apart more than 5 Å (cutoff range for magnetic interactions). (ii) If two magnetic sites α and β (not necessarily belong to the same supercell) are within 5 Å, then the distance between α and β′, a periodic image of β (β′ ≠ β) is longer than 5 Å except when α-β and α-β′ are symmetrically equivalent. Within the Ising model, the total energy of the supercell (*E*) can be expressed as follows:

1 $$E={E}_{0}+\mathop{\sum }\limits_{I}^{m}\,{J}_{I}{\rm{(}}{N}_{I,{\rm{P}}}-{N}_{I,{\rm{A}}}{\rm{)}},$$

where *E*_0_ is the base energy excluding the magnetic interaction, and *I* is the index for independent exchange interactions (total *m* interactions) with the maximum range of 5 Å and the exchange parameter of *J*_*I*_. In Eq. (1), *N*_*I*,P_ and *N*_*I*,A_ are the numbers of parallel and antiparallel spin pairs within the supercell corresponding to the interaction *I*, respectively. Then, based on the ferromagnetic configuration (all spin-up), diverse spin configurations are sampled by spin-flipping a magnetic pair (both atoms) or a certain magnetic site. The number of resulting equations is larger than *m* and an optimal {*E*_0_, *J*_*I*_} can be obtained by the pseudoinverse method. We find that this approach produces essentially the same parameters as the original scheme but is more reliable and efficient.

### Selection of materials

**Fig. 1:**
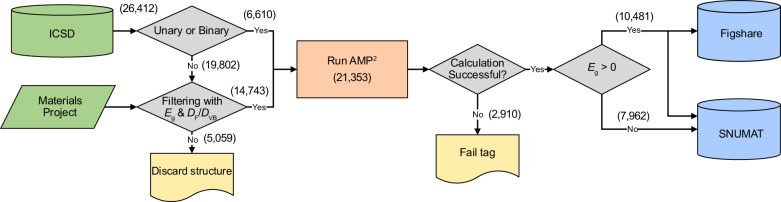
The computational workflow for collecting the dataset. Numbers in the parentheses indicate material counts.

Figure 1 schematizes the workflow of constructing the database. Starting from the ICSD, we only consider compounds consisting of elements with atomic number (*Z*) < 84. Among the lanthanides, we limit the elements to La and Ce. We remove structural duplicates and structures with partially occupied sites, and also omit large primitive cells that contain more than 40 atoms. For unary and binary compounds, all the structures are calculated with AMP^2^. For ternary and higher compounds, we utilize information on *E*_g_ and DOS in the Materials Project database (calculated by PBE) to filter out materials that are likely to be metallic or large-gap insulators. To be specific, we exclude materials with ${E}_{{\rm{g}}}^{{\rm{GGA}}}$ bigger than 3 eV since they are likely to have ${E}_{{\rm{g}}}^{{\rm{HSE}}}$ larger than 5 eV. (Compiling data of 4,421 compounds from the previous screening studies^24,29–31^, we find that 99.7% of materials with ${E}_{{\rm{g}}}^{{\rm{HSE}}}$ < 5 eV have ${E}_{{\rm{g}}}^{{\rm{GGA}}}$ < 3 eV.) We also include metallic materials with *D*_F_/*D*_VB_ < 0.8 for possible gap opening (see above; a larger threshold is used because of low-resolution DOS in the Materials Project). If a Materials Project data has incomplete entries for *E*_g_ or DOS, the material is included in the computation list. In this way, we could factor out 5,059 materials from the list of ternary and higher compounds. Finally, we calculate 21,353 materials with AMP^2^. After computation, we collect 10,481 materials with finite *E*_g_ (unary: 63, binary: 1,919, ternary: 5,074, quaternary: 2,804, quinary: 573, and higher: 48).

## Data Records

All the calculated properties for 10,481 compounds can be downloaded from the Figshare Repository^17^. The whole data including metals are also uploaded to SNUMAT (www.snumat.com), which provides easy search and visualization of materials through its own interactive interface. SNUMAT also supports REST API^32^ for users to search the materials with authorization. The authorization token expires 24 hours after they are issued.

### File format

The data are stored in the JSON format. The name of the file is *X*_ICSD#.json, where *X* is chemical formula and ICSD# is the ICSD collection code of the initial structure used for calculation. Each JSON file includes final relaxation structure information, ${E}_{{\rm{g}}}^{{\rm{GGA}}}$, ${E}_{{\rm{g}}}^{{\rm{HSE}}}$, and DOS. Table 1 summarizes keys for metadata.

**Table 1:** Description of metadata keys in JSON file.

Key	Type	Description
SNUMAT_id	string	ID in the SNUMAT
ICSD_number	int	ICSD collection code
Band_gap_GGA	float	Calculated fundamental band gap in GGA (eV)
Band_gap_GGA_optical	float	Calculated direct band gap in GGA (eV)
Band_gap_HSE	float	Calculated fundamental band gap in HSE (eV)
Band_gap_HSE_optical	float	Calculated direct band gap in HSE (eV)
Direct_or_indirect	string	Type of band gap in GGA (direct or indirect)
Direct_or_indirect_HSE	string	Type of band gap in HSE (direct or indirect)
Structure_rlx	string	Relaxed structure information (VASP POSCAR format)
Space_group_rlx	int	Space group number of relaxed structure
Magnetic_ordering	string	Magnetic ordering of final structure
SOC	boolean	Spin-orbit coupling (True or False)

### Graphical representation of the data

**Fig. 2:**
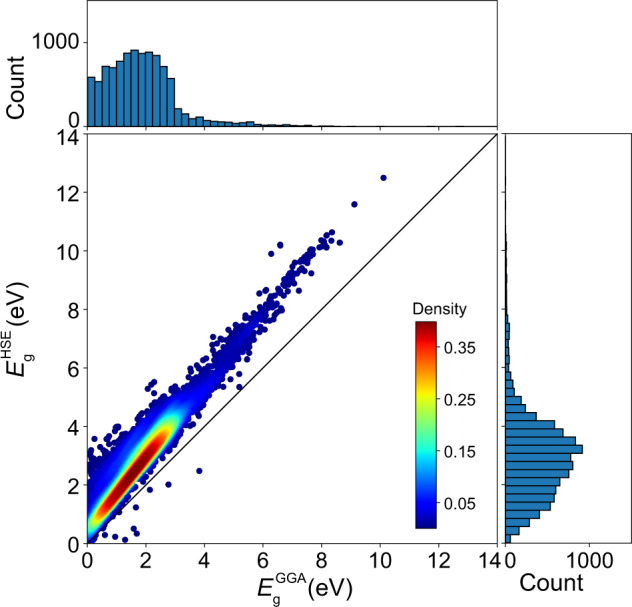
Distribution of ${E}_{{\rm{g}}}^{{\rm{GGA}}}$ and ${E}_{{\rm{g}}}^{{\rm{HSE}}}$. Top and right are occurrence histograms of ${E}_{{\rm{g}}}^{{\rm{GGA}}}$ and ${E}_{{\rm{g}}}^{{\rm{HSE}}}$, respectively.

In Fig. 2, we present the distribution of ${E}_{{\rm{g}}}^{{\rm{GGA}}}$ and ${E}_{{\rm{g}}}^{{\rm{HSE}}}$ for 10,481 materials. Most materials with ${E}_{{\rm{g}}}^{{\rm{HSE}}}$ > 5 eV (663 cases) are unary or binary compounds for which AMP^2^ is applied to the whole structure dataset from ICSD.

## Technical Validation

### Comparison to experimental measurements and other databases

**Fig. 3:**
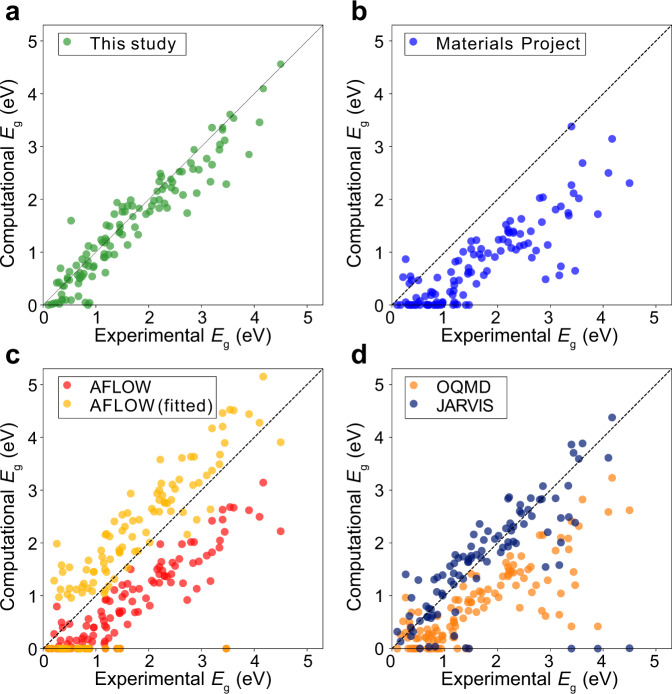
Comparison of *E*_g_ for benchmark materials between experimental and theoretical data from (**a**) this work, (**b**) Materials Project, (**c**) AFLOW and (**d**) OQMD and JARVIS. AFLOW-fitted values are obtained from ${E}_{{\rm{g}}}^{{\rm{f}}{\rm{i}}{\rm{t}}}=1.34\,{E}_{{\rm{g}}}^{{\rm{G}}{\rm{G}}{\rm{A}}}+0.913$ eV.

In Fig. 3a, we compare experimental and theoretical values for 116 benchmark materials with experimental *E*_g_ between 0 and 5 eV. The list of compounds is shown in Online-only Table 1. For comparison, theoretical results from other databases are also shown in Fig. 3b–d. The RMSE values are 0.36 eV (present work), 1.05 eV (Materials Project), 0.93 eV (AFLOW), 0.75 eV (AFLOW fitted), 1.02 eV (OQMD), and 0.85 eV (JARVIS). (The meta-GGA values of 19 materials, mostly with small *E*_g_, are missing in JARVIS.) This confirms that the present database provides more accurate *E*_g_ than the existing databases on average. In particular, we correctly identify the semiconducting nature for small-gap semiconductors such as AgSbTe_2_, CdO, CoP_3_, Cu_3_AsSe_4_, Cu_3_SbS_4_, Cu_3_SbSe_4_, CuFeS_2_, Ge, Mg_2_Sn, RhSb_3_, and ZnSnSb_2_, which are mostly misreported as metals in other databases. In addition, other databases exhibit pronounced errors for every antiferromagnetic material (CuFeS_2_, CuO, FeF_2_, MnO, MnTe, and NiO) because these materials are considered as ferromagnetic or non-magnetic. (For non-magnetic materials in Online-only Table 1, the *E*_g_ calculated with pure PBE (without +*U* and SOC) by AMP^2^ agrees well with those from Materials Project (the mean absolute error is 0.034 eV).)

In most cases, the present database provides *E*_g_ that agrees well with experiment. However, there are some materials with large errors of ≥0.5 eV such as AgAlTe_2_, Cu_3_AsSe_4_, CuAlSe_2_, CuBr, CuCl, CuFeS_2_, CuO, Ge, IrSb_3_, La_2_S_3_, MnO, RhAs_3_, RhSb_3_, SnO_2_, SrS, and ZnO. For small-gap materials such as Cu_3_AsSe_4_, Ge, and IrSb_3_, *E*_g_ is sensitive to the lattice parameters that are slightly overestimated by PBE. Employing experimental lattice parameters or those relaxed within HSE significantly improves the results^15^. For Cu-bearing materials, it is known that HSE often exhibits substantial errors in *E*_g_ due to nonlocal screening effects in Cu, which requires *GW* calculations^33,34^. We also note that van der Waals interactions are not described by semilocal functionals, and lattice parameters can be overestimated in layered structures such transition-metal dichalcogenides^35^. This can significantly affect *E*_g_, and so care is needed in referring to *E*_g_ in layered materials. The present results do not consider finite-temperature effects on *E*_g_, which can be significant in some materials, for example, hybrid perovskites^36^. More generally, *E*_g_ dataset with the ultimate theoretical accuracy would be obtained by the quasiparticle approaches such as *GW* or Bethe-Salpeter equations^37,38^.

## Data Availability

The AMP^2^ package used for constructing the present database is available at https://github.com/MDIL-SNU/AMP2 and was released under a GPLv3 (GNU General Public License). The package requires pre-installation of numpy, scipy, spglib, and PyYAML modules. Detailed guidelines and examples can be found in the manual (https://amp2.readthedocs.io/en/latest/).

## Online-only Table

**Online-only Table 1:** The list of materials and their band gaps from experiment, present work, Materials Project, OQMD, AFLOW, AFLOW (fitted), and JARVIS.

Name	ICSD	Space group	Exp.	Band gap					
				Present work	Materials Project	OQMD	AFLOW	AFLOW (fitted)	JARVIS
Mg_2_Pb	104846	225	0.1	0	0	0	0	0	0.32
InSb	640411	216	0.16	0.01	0	0.26	0	0	0.13
Bi_2_Te_3_	15753	166	0.21	0.02	0.53	0.52	0.28	1.29	-
PbTe	63098	225	0.26	0.42	0.87	0.97	0.8	1.99	1.4
CdSnAs_2_	16737	122	0.26	0.04	0.05	0.3	0	0	-
Sb_2_Te_3_	193341	166	0.3	0.43	0.01	0.21	0.04	0.97	-
Cu_3_SbSe_4_	400652	121	0.31	0.11	0	0	0	0	-
Te	161690	152	0.33	0.69	0.41	0.5	0.15	1.12	0.62
Bi_2_Se_3_	617072	166	0.35	0.23	0.54	0.42	0.49	1.58	-
Mg_2_Sn	104869	225	0.36	0.09	0	0	0	0	0.24
InAs	610687	216	0.36	0.1	0	0.37	0	0	0.4
PbSe	63096	225	0.37	0.36	0.47	0.69	0.4	1.45	0.96
ZnSnSb_2_	42669	122	0.4	0.36	0	0	0	0	0.32
CoP_3_	624594	204	0.43	0.11	0	0	0	0	-
CdSb	52830	61	0.46	0.52	0.14	0.19	0.09	1.03	-
Cu_3_SbS_4_	2857	121	0.46	0.32	0	0	0	0	-
PbS	62190	225	0.5	0.47	0.47	0.69	0.47	1.54	1.3
ZnSb	43265	61	0.5	0.63	0.04	0.2	0.17	1.14	-
CuFeS_2_	60166	122	0.53	1.6	0	0	0	0	0
CdGeAs_2_	153593	122	0.53	0.09	0.08	0.34	0.03	0.96	0.52
CoSb_3_	153504	204	0.63	0.75	0.17	0.22	0.15	1.12	-
ZnSnAs_2_	18203	122	0.65	0.55	0.01	0.2	0	0	0.8
Ge	41980	227	0.67	0.17	0	0	0	0	0.61
GaSb	41675	216	0.67	0.41	0	0.37	0	0	0.59
CoAs_3_	31111	204	0.69	0.52	0	0.15	0	0	-
InN	109463	186	0.7	0.5	0	0.28	0	0	0.76
Mg_2_Ge	52283	225	0.74	0.54	0.19	0.24	0.19	1.17	0.58
TlSe	44706	140	0.74	0.55	0.25	0	0.11	1.06	0.74
Mg_2_Si	104864	225	0.77	0.59	0.24	0.28	0.24	1.23	0.62
RhSb_3_	650248	204	0.8	0.16	0	0.15	0	0	-
CdO	181294	225	0.8	0.76	0	0	0	0	1.31
CuGaTe_2_	28738	122	0.82	0.96	0.2	0.4	0.43	1.49	0.04
RhAs_3_	34052	204	0.85	0	0	0	0	0	-
ZnGeAs_2_	16735	122	0.85	0.84	0.05	0.34	0.17	1.14	1.07
HgIn_2_Te_4_	25652	82	0.86	1.12	0.52	0.61	0.56	1.67	1.35
CuInSe_2_	73351	122	0.86	0.74	0.02	0.16	0	0	0.64
Cu_3_AsSe_4_	610361	121	0.88	0.05	0	0	0	0	-
Zn_3_As_2_	44091	137	0.93	0.54	0	0	0.13	1.09	-
CuInTe_2_	169048	122	0.95	0.71	0.02	0.24	0.25	1.25	1.04
AgInSe_2_	28751	122	0.96	0.74	0.06	0.16	0.4	1.45	1
CuGaSe_2_	247513	122	0.96	1.08	0.04	0.25	0.4	1.45	1.39
AgGaSe_2_	28748	122	1.1	1.24	0.25	0.41	0.7	1.86	1.56
Si	51688	227	1.12	1.19	0.62	0.77	0.61	1.74	1.28
AgGaTe_2_	28749	122	1.15	0.9	0.19	0.32	0.52	1.62	-
Cu_2_CdSnS_4_	238144	121	1.16	0.97	0	0.21	0.1	1.05	-
CdSnP_2_	22183	122	1.17	0.9	0.27	0.51	0.14	1.1	1.21
InSe	185172	194	1.17	1.18	0.46	0.55	0.43	1.5	1.43
IrSb_3_	640958	204	1.18	0.61	0.05	0.21	0	0	0.24
AglnS_2_	28750	122	1.18	1.26	0.38	0.49	0.92	2.16	1.62
ZnIn_2_Te_4_	25650	82	1.2	1.51	0.82	0.92	0.93	2.17	1.77
CuInS_2_	66865	122	1.2	1.22	0.01	0.42	0.4	1.45	0.69
Cu_3_AsS_4_	14285	31	1.24	0.96	0.01	0	0.2	1.18	-
Cdln_2_Te_4_	25651	82	1.26	1.49	0.84	0.92	0.9	2.12	1.72
InP	53105	216	1.27	1.12	0.47	0.74	0.58	1.69	1.39
GaAs	41981	216	1.35	0.95	0.19	0.57	0.3	1.32	1.32
ZnGa_2_Te_4_	290911	82	1.35	1.72	1.01	1.18	1.11	2.41	1.74
CuO	16025	15	1.4	1.94	0	0	0	0	0.01
CdTe	93944	216	1.44	1.63	0.59	0.87	0.71	1.87	1.64
HgIn_2_Se_4_	25649	82	1.45	1.38	0.62	0.72	0.67	1.81	1.64
ZnSnP_2_	22179	122	1.45	1.53	0.7	0.92	0.68	1.83	1.69
MnTe	174028	194	1.46	1.47	0	0	0	0	0
BAs	43871	216	1.5	1.86	1.21	1.42	1.2	2.53	1.93
Se (gray)	164264	152	1.5	1.76	1.06	0.88	0.98	2.23	1.71
CdTe	620518	186	1.5	1.67	0.62	0.87	0.71	1.92	1.76
Cdln_2_Se_4_	151954	82	1.55	1.77	0.95	1.03	1.04	2.31	2.16
CdSiAs_2_	22187	122	1.6	1.14	0.4	0.69	0.46	1.53	1.33
AlSb	44325	216	1.6	1.88	1.23	1.38	1.23	2.57	1.78
AgGaS_2_	23698	122	1.66	1.99	0.96	1.09	1.5	2.94	2.36
GaTe	635512	12	1.69	1.88	1.06	0.93	0.89	2.12	1.72
ZnSiAs_2_	22184	122	1.7	1.67	0.89	1.06	1.12	2.42	1.85
CdSe	41826	186	1.74	1.47	0.57	0.77	0.69	1.84	2.06
CdGeP_2_	100467	122	1.8	1.33	0.64	0.9	0.7	1.85	1.65
ZnIn_2_Se_4_	25647	82	1.82	1.83	0.98	1.12	1.13	2.44	2.22
HgGa_2_Se_4_	188545	82	1.95	1.72	0.91	1.05	0.95	2.2	2.08
GaSe	63122	194	2.02	1.77	1.23	0.97	0.81	2.01	2.14
CuAlTe_2_	28735	122	2.06	1.88	1.02	1.2	1.29	2.65	1.82
BP	615154	216	2.1	1.97	1.24	1.37	1.25	2.59	1.91
AlAs	185081	216	2.16	2.15	1.52	1.63	1.5	2.94	2.28
ZnGeP_2_	16734	122	2.2	1.83	1.19	1.37	1.25	2.59	1.93
CdSiP_2_	22188	122	2.2	1.99	1.43	1.4	1.41	2.81	2.02
ZnGa_2_Se_4_	44887	82	2.2	2.34	1.39	1.54	1.55	3	2.84
AgI	164963	216	2.22	2.49	1.37	1.48	1.98	3.58	2.87
GaP	77087	216	2.24	2.43	1.59	1.75	1.64	3.13	2.37
ZnTe	185141	216	2.26	2.19	1.08	1.45	1.48	2.9	2.23
ZnSiP_2_	22190	122	2.3	1.92	1.36	1.44	1.38	2.77	2.03
β-SiC	603798	216	2.3	2.26	1.39	1.59	1.37	2.76	2.31
AgAlTe_2_	28746	122	2.35	1.84	1.05	1.2	1.36	2.75	1.99
CdS	154186	186	2.42	2.38	1.12	0.77	1.25	2.6	2.6
CdGa_2_Se_4_	2287	82	2.43	2.22	1.35	1.45	1.41	2.82	2.72
AlP	24490	216	2.45	2.33	1.63	1.75	1.63	3.11	2.56
AgAlSe_2_	28745	122	2.5	2.17	1.14	1.33	1.56	3.02	2.36
ZnSe	185134	216	2.58	2.54	1.17	1.5	1.7	3.2	2.63
La_2_S_3_	15151	62	2.73	1.75	1.03	1.2	0.92	2.16	-
AgI	62789	186	2.63	2.52	1.4	1.55	1.98	3.61	2.85
CuAlSe_2_	28734	122	2.65	2.08	0.9	1.07	1.24	2.58	2.51
BeTe	53945	216	2.8	2.69	2.02	2.15	2.02	3.63	2.82
HgGa_2_S_4_	189737	82	2.84	2.57	1.57	0.72	1.62	3.1	2.83
α-SiC	164429	186	2.86	2.94	2.04	2.15	2.02	3.63	3.08
CuBr	23989	216	2.91	2.12	0.49	0.62	1.13	2.44	1.55
Cul	33724	216	2.95	2.56	1.14	1.29	1.65	3.14	2.2
Al_2_Se_3_	14373	9	3.1	2.77	1.81	1.86	1.76	3.28	2.85
CuCl	23988	216	3.17	2.33	0.56	0.64	1.28	2.64	1.58
CeO_2_	184584	225	3.2	3.36	1.87	2.1	2.42	4.17	2.01
ZnO	154486	186	3.2	2.66	0.73	1.04	1.82	3.37	2.47
GaN	153890	186	3.34	2.94	1.74	2.09	1.91	3.49	3.08
CuAlS_2_	189083	122	3.35	3.04	1.69	1.88	2.05	3.67	2.49
FeF_2_	14143	136	3.4	3.63	3.38	0.42	2.63	4.46	0
ZnGa_2_S_4_	44886	82	3.4	3.31	2.27	2.41	2.44	4.2	3.86
CdGa_2_S_4_	106362	82	3.44	3.12	2.12	1.45	2.21	3.89	3.71
SnO_2_	154960	136	3.47	2.29	0.65	1.2	0	0	2.39
ZnS	77090	216	3.54	3.61	2.02	2.32	2.68	4.52	3.59
BeSe	616419	216	3.61	3.54	2.69	2.82	2.67	4.51	3.88
MnO	9864	225	3.9	2.85	1.72	0.42	2.62	4.44	0
SrS	651054	225	4.1	3.46	2.5	2.59	2.5	4.28	3.61
BeS	44724	216	4.17	4.1	3.15	3.23	3.14	5.15	4.38
NiO	43740	225	4.5	4.53	2.31	2.62	2.22	3.91	0.01
RMSE				0.36	1.05	1.02	0.93	0.75	0.85

The experimental data were adopted from a CRC Handbook^39^ except AgAlSe_2_^40^, AgAlTe_2_^40^, AgGaTe_2_^40^, CdO_2_^41^, CdSnP_2_^42^, CuAlS_2_^43^, CuAlTe_2_^44^, HgIn_2_Se_4_^45^, and InN^46^ for which more reliable measurements are cited, and CeO_2_^47^ and La_2_S_3_^48^ which represent lanthanides.
